# The significance of linoleic acid in food sources for detritivorous benthic invertebrates

**DOI:** 10.1038/srep35785

**Published:** 2016-10-21

**Authors:** J. Arie Vonk, Bernd F. van Kuijk, Mick van Beusekom, Ellard R. Hunting, Michiel H. S. Kraak

**Affiliations:** 1 Department of Aquatic Environmental Ecology, Institute for Biodiversity and Ecosystem Dynamics (IBED), University of Amsterdam, Sciencepark 904, 1098 XH Amsterdam, The Netherlands; 2 Institute of Environmental Sciences (CML), Leiden University, PO Box 9518, 2300 RA Leiden, The Netherlands

## Abstract

Chemical composition of organic matter (OM) is a key driver for detritus consumption by macroinvertebrates and polyunsaturated fatty acid (PUFA) content is considered a candidate indicator of food palatability. Since traditionally used complex natural OM covaries in many quality attributes, it remains uncertain whether benthic invertebrates developed an actual preference for PUFA-rich food. Therefore we aimed to test the influence of the PUFA linoleic acid on OM consumption by aquatic macroinvertebrates using standardized surrogate substrates (decomposition and consumption tablet, DECOTAB) with added linoleic acid (PUFA) in comparison to consumption of DECOTAB containing only cellulose (Standard) or ground macrophytes (Plant). In microcosms, we observed a higher consumption rate of PUFA DECOTAB in comparison to Standard DECOTAB in two functionally distinct invertebrate species (*Lumbriculus variegatus* and *Asellus aquaticus*). This effect appeared to be overruled in the field due to unknown sources of natural variation. Although we observed higher consumption rates in species-rich ditches compared to species-poor ditches, consumption rates were comparable for all three types of DECOTAB deployed. Upon reduced food quality and palatability, results presented here hint that PUFA like linoleic acid may be a key OM attribute driving the performance of benthic macroinvertebrates and inherent functioning of aquatic ecosystems.

Dead organic matter (OM) fuels benthic food webs^1^,^2^ by serving as a food source for a diverse array of microorganisms^3^,^4^ and macroinvertebrates^5^,^6^. The functional diversity of benthic invertebrates positively influences OM-degradation and thereby the functioning of aquatic ecosystems^7^,^8^, suggesting close links between the diversity of detritivorous invertebrates and the processing of OM^9^. Degradation of aquatic ecosystems and inherent impoverishment of macroinvertebrate communities^10^, could thus strongly affect litter consumption and processing of OM, ultimately leading to poorly functioning systems.

The chemical composition of OM is a key driver that can either promote or retard OM-degradation and the availability of newly produced, high quality OM can cause strong growth responses in invertebrates^11^,^12^,^13^,^14^,^15^. It is often argued that polyunsaturated fatty acid (PUFA) content of food items such as autochthonous plant litter, hyphomycetes, and particulate sediment organic matter offers a candidate indicator of food quality for decomposers^13^,^16^,^17^ and is a key factor modulating the productivity of primary consumers^18^,^19^. Besides the well studied omega-3 PUFA^20^, omega-6 PUFA are essential in aquatic food webs for the development and growth of invertebrates, which is also reflected in the low omega-3:omega-6 ratios in many benthic detritivores^21^,^22^. The omega-6 linoleic acid (18:2 n-6) is commonly considered an essential food component for many macroinvertebrates as they cannot synthesize this PUFA de novo^23^,^24^. It is the precursor of the essential PUFA arachidonic acid (20:4 n-6) and is produced by plants and green algae^25^. Linoleic acid can be a substantial component of the PUFA content of sediments in aquatic systems like lowland ditches^26^, related to a high input of relatively omega-6 rich leaf litter from terrestrial plants^27^. Many studies indeed have provided indications that OM-PUFA content could be an essential OM-attribute that influences behaviour of macroinvertebrates^13^,^14^,^23^,^28^. However, observed positive relations between OM PUFA content and invertebrate preferences rely on OM substrates that contain many confounding attributes^29^ and thus experimental studies manipulating PUFA specifically within OM substrates are required^30^,^31^,^32^.

Since it can be expected that consumption of OM by species-rich detritivorous invertebrate communities more strongly depends on OM quality compared to consumption by species-poor communities due to preferences of individual species for higher quality detritus sources, we hypothesize that addition of linoleic acid to standardized OM (Decomposition and Consumption tablet; DECOTAB^32^) enhances consumption by macroinvertebrate species and that consumption rates will become more comparable to natural litter. To test this assumption, this study aimed to assess the significance of linoleic acid, as an example of an essential PUFA, for OM consumption by aquatic macro-invertebrates, both in laboratory microcosms and under natural conditions. We compared consumption of DECOTAB that contained standardized concentrations of linoleic acid to consumption of DECOTAB containing only cellulose substrate (low food quality) and DECOTAB containing ground macrophytes (high food quality). These OM treatments were offered to two functionally distinct invertebrate species (the deposit feeder *Lumbriculus variegatus* and the shredder *Asellus aquaticus*) in laboratory microcosms and to natural invertebrate communities in lowland ditches with either a species-rich (>10 taxa) or species-poor (<5 taxa) community composition.

## Results

### Consumption in laboratory microcosms

**Figure 1:**
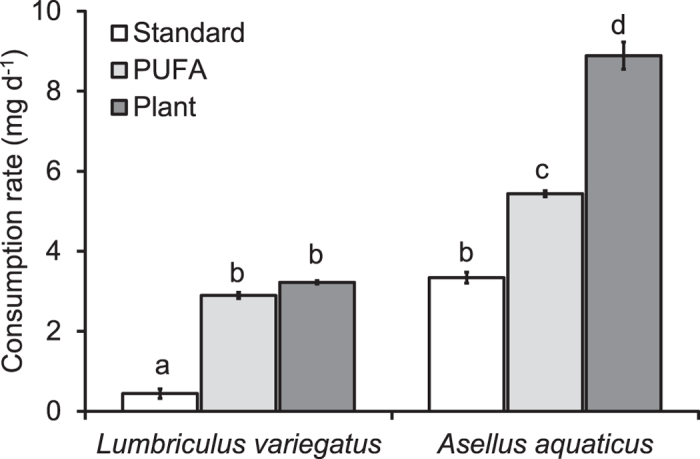
Average (±SE) consumption rates (mg d^−1^) of the three DECOTAB types by *L. variegatus* and *A. aquaticus* in microcosms over sixteen days (*n* = 5). Letters indicate significant differences between consumption rates (ANOVA F_5,24_ = 305, P < 0.001, Post-hoc Tukey’s b test).

In the control microcosms, none of the three DECOTAB types showed significant mass loss related to leaching after 16 days of exposure (P > 0.50). Consumption rates were significantly different between species and DECOTAB qualities (ANOVA: F_5,24_ = 305, P < 0.001). The shredder *A. aquaticus* consumed all DECOTAB types significantly faster than the deposit feeder *L. variegatus* (P < 0.001; Fig. 1). DECOTAB quality significantly influenced consumption rates by both macroinvertebrates (P < 0.001). Standard DECOTAB, containing only cellulose as food source, were consumed significantly less compared to PUFA and Plant DECOTAB. *A. aquaticus* consumed Plant DECOTAB faster compared to PUFA DECOTAB, while *L. variegatus* consumed PUFA and Plant planDECOTAB at comparable rates (Fig. 1).

### Consumption in ditches

From the cages, almost 1700 macroinvertebrates were collected and identified, per cage on average 31 ± 3 individuals (mean ± SE) belonging to 4.9 ± 0.2 taxa. The dominant taxa were *Valvata* spp. (average 10.4 individuals cage^−1^), *Chironomus* spp. (9.1) and *Gammarus pulex* (4.6). Comparable macroinvertebrate numbers were collected from cages in both type of ditches, however, a significant difference in functional feeding composition of the macrofaunal communities was observed between species-rich and species-poor ditches (Gower-based ANOSIM, P = 0.046). Species-poor ditches were characterized by a dominance of deposit feeders (41.8% of the community versus 20.7% in species-rich ditches) and low representation of shredders (16.5% and 26.6%, respectively). Scrapers occurred in comparable densities in both types of ditches (34.7% and 37.0%, respectively). Predators and filter-feeders occurred in low densities.

**Figure 2:**
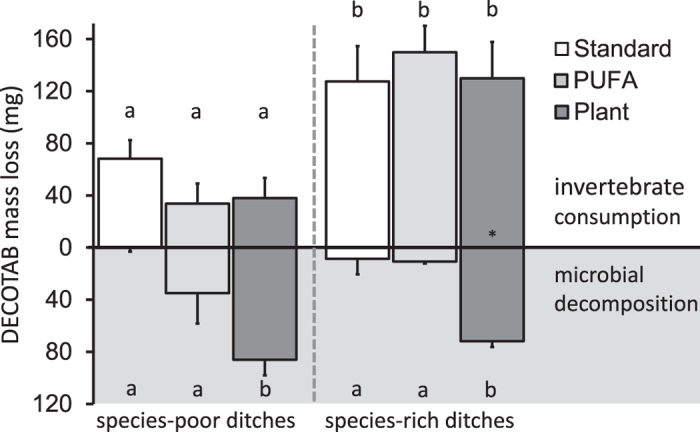
Loss of DECOTAB mass (mg) per cage after 43 days due to invertebrate consumption and microbial decomposition (note inverse scaling for latter) in species-poor and species-rich ditches (mean ± SE, consumption *n* = 9; decomposition *n* = 3). Significant differences in DECOTAB loss between ditches due to invertebrate consumption (upper part) and decomposition (lower part) are indicated by letters. *n = 8.

In the field, degradation (total of macroinvertebrate consumption and microbial decomposition) after 43 days was different between ditch species richness and between DECOTAB qualities. Degradation in species-rich ditches was faster compared to species-poor ditches (ANOVA F_1,47_ = 16.1, P < 0.001). Degradation of Standard DECOTAB (in both species-poor and species-rich ditches, 68 ± 14 mg and 136 ± 27 mg, mean ± SE respectively) was significantly slower than degradation of Plant DECOTAB (124 ± 15 mg and 201 ± 28 mg, respectively), while for PUFA DECOTAB degradation (69 ± 15 mg and 160 ± 20 mg, respectively) was intermediate in species-rich ditches (ANOVA F_2,47_ = 4.53, P = 0.016). Microbial decomposition of DECOTAB (fine mesh cages) was comparable between ditches with different species richness (P = 0.78), but was significantly different between DECOTAB qualities (F_2,12_ = 21.2, P < 0.001; Fig. 2). Microbial decomposition of Standard- and PUFA DECOTAB was lower (4.3 ± 4.1 mg d^−1^ and 22.6 ± 11.8 mg d^−1^, respectively) than decomposition of Plant DECOTAB (78.6 ± 6.6 mg d^−1^). Macroinvertebrate consumption was significantly higher for all DECOTAB qualities in the species-rich ditches compared to the species-poor ditches (ANOVA F_2,47_ = 25.5, P < 0.001), but consumption was similar for all DECOTAB qualities within each ditch type (P = 0.56; Fig. 2).

## Discussion

Adding specific hydrophobic compounds to the agar matrix of the DECOTAB, like linoleic acid or other PUFA, provided opportunities to specifically modify food quality of this artificial substrate. In this way, the effects of a single compound or of mixtures of compounds on food palatability and consumption rates could be assessed and compared between various detritivorous organisms. Here we determined the influence of food quality on consumption rates by benthic macroinvertebrates, demonstrating the importance of food PUFA content for detritivores under controlled conditions. We observed a higher consumption of linoleic acid enriched OM (PUFA DECOTAB) in comparison to cellulose only OM (Standard DECOTAB), indicating that the addition of linoleic acid to food sources improved the OM quality for the detritivorous macroinvertebrates tested in this study. The observed importance of PUFA content for consumption rate corroborates previous research in the field, where PUFA content was shown to be a good indicator of food quality for invertebrates^14^,^23^,^33^ and enhanced growth rates of pelagic and benthic macroinvertebrates^13^,^18^. The presently observed importance of PUFA for OM-consumption is likely caused by the inability of both *A. aquaticus* and *L. variegatus*, like most invertebrates, to synthesize linoleic acid de novo^23^,^24^. Although it remains uncertain whether our results reflect the significance of PUFA for all detritivorous macroinvertebrate species on ecologically relevant spatial and temporal scales, they do indicate that PUFA can indeed become a key attribute governing consumption of the available OM.

In our laboratory experiment we tested two functionally distinct invertebrate species for their consumption rates of different quality food sources. Besides expected differences in consumption rates between species, with the shredder *A. aquaticus* consuming all DECOTAB qualities significantly faster than the deposit feeder *L. variegatus*, we observed large differences in the relative influence of litter quality on consumption rates by both detritivorous species. This suggests that the significance of PUFA on OM-consumption is different between species. In comparison to consumption rate of surrogate litter consisting of only cellulose (Standard DECOTAB), surrogate litter consisting of cellulose and additional 5‰ dry mass linoleic acid was consumed by *A. aquaticus* at 163% and by *L. variegatus* even at 652%. For *A. aquaticus*, the consumption rate of linoleic enriched standardized OM (cellulose) was around 60% of complex plant OM (Plant DECOTAB), while for *L. variegatus* the addition of linoleic acid to standard OM resulted in comparable consumption rates (~90%) compared to consumption of complex plant OM. The latter suggests that linoleic acid is an important component governing OM consumption by *L. variegatus.*

Although we only tested two species that represent two different feeding habits, it can thus be speculated that different responses to food quality between functional feeding groups can have impact on organic matter processing by the detritivorous macroinvertebrate community. Detritivorous macroinvertebrates belonging to different functional feeding groups, e.g. shredders and deposit feeders^34^, consume different fractions of organic matter and are therefore expected to react directly to variations in food quality. This is further depending on the potential for bioconversion of linoleic acid to long-chain PUFA such as arachidonic acid and/or the ability to directly take up long-chain PUFA from food sources by macroinvertebrates^35^. In ecologically degraded ditches, communities are not only characterized by lower densities of macroinvertebrates, but also profound changes in functional composition of these communities^10^,^26^. Alteration of the functional composition of macroinvertebrate communities (due to anthropogenic pressures) and chemical composition of the OM (due to e.g. land use change) could thus likely cause changes in consumption rates of different quality organic matter resources and inherent energy fluxes through aquatic food webs^36^.

In this study we observed higher consumption rates in lowland ditches with a more diverse macroinvertebrate community, comparable to various aquatic systems in which species richness and trophic diversity positively affected decomposition^7^,^8^. However, decomposition and consumption of the different qualities of the litter surrogates in the ditches only partly reflected the observations made in the controlled microcosm experiment. In species-rich ditches, degradation of the DECOTAB with added linoleic acid was not significantly lower compared to DECOTAB that contained ground macrophytes, however, in all ditches the degradation of PUFA DECOTAB was comparable to cellulose only (Standard) DECOTAB. For microbial decomposition we observed differences between litter qualities, with 48% of the total loss of the Plant DECOTAB accounted for by microbial decomposition contrary to relatively low losses for Standard and PUFA DECOTAB (4% and 20%, respectively). Macroinvertebrate consumption rates were comparable for all three types of DECOTAB deployed in each of the ditch types. Hence, in our field experiment the influence of surrogate litter quality was overruled by the heterogeneity in invertebrate community composition and other sources of natural variation.

One of the sources of natural variation may have been the availability of other food sources which could have influenced consumption of the introduced artificial substrates at the ditch scale. Detritivorous macroinvertebrates experience large shifts in food quality over the seasons^37^,^38^ due to leaf litter decay^27^, fungal colonization of litter^17^ and the changes in relative contribution of OM sources (PUFA-rich autochthonous algal detritus versus PUFA-poor allochthonous tree leaf litter) to the available food during the year^39^. This also results in changes in omega-3:omega-6 ratios of PUFA in OM available for benthic invertebrates^21^. Adaptations to shifts in food quality can be observed by differences in growth response and PUFA accumulation in benthic detritivores like *A. aquaticus* between seasons, due to season-specific physiological status^40^. In degraded peatland ditches other factors besides food quality of detrital sources can also be limiting for macroinvertebrate communities, such as availability of suitable habitat substrate^26^. Hence, while PUFA can be key to the performance of invertebrates under controlled, food limiting conditions, its significance can be fading in natural heterogenic environments where other factors (e.g. habitat deterioration) more strongly drive macroinvertebrate communities.

In conclusion, by using surrogate litter in which we manipulated PUFA content and food palatability, we observed a strong influence of the presence of the PUFA linoleic acid in food items on OM consumption rates by aquatic invertebrates in our laboratory microcosms. Observed differences in consumption rates between the two species are most likely caused by differences in feeding mode and preferences for OM in different stages of decomposition. However, the observed influence of linoleic acid on OM consumption was overruled in a natural environment, most likely caused by heterogeneity in invertebrate community composition and other sources of natural variation. Results presented here thus hint that omega-6 PUFA like linoleic acid may be a key OM attribute driving the performance of macroinvertebrates in benthic environments where food palatability is drastically reduced.

## Methods

### DECOTAB preparation

Surrogate litter sources that have traditionally been used to study invertebrate consumption are difficult to manipulate chemically to study effects of specific compounds^29^,^30^,^31^. The recently developed DECOTAB^32^, consisting of cellulose in an agar matrix, offers an opportunity to overcome these constraints, since their composition can be altered by adding natural organic matter^41^, plant litter or specific substances like the PUFA linoleic acid. We studied macroinvertebrate consumption and microbial decomposition using three types of DECOTAB: Standard, PUFA, and Plant. Standard DECOTAB were prepared following the procedures of Kampfraath *et al*.^28^. The solution to mould DECOTAB contained 60 g L−1 of powdered cellulose (Sigma– Aldrich, St. Louis, Missouri), 20 g L−1 of purified agar (Oxoid Ltd., Basingstoke Hampshire) and 60 μmol L−1 ascorbic acid as antioxidant (Merck GmbH, Darmstadt) dissolved in deionized water. For PUFA DECOTAB, 0.40 g L−1 linoleic acid (99% GC, FLUKA, Rhine Valley) was added. This corresponded to 5 mg PUFA g−1 dry matter, in accordance with the linoleic acid content of submerged plants^42^. In Plant DECOTAB, the cellulose was substituted by powdered macrophytes consisting of the submerged species *Potamogeton pusillus* and *Myriophyllum spicatum* to represent a natural litter source. In order to minimize microbial resources as potential food source in the Plant DECOTAB, fresh plant material was collected, rinsed and dried at 60 °C. Plant DECOTAB contained the most balanced food source including a wide range of PUFA^16^,^43^ and represented consumption of natural and high quality OM. To prepare each type of DECOTAB, the agar was boiled for 3 min and cooled down under continuous stirring to 60 °C at which point the other compounds were added. The mixture was poured into polycarbonate moulds (15 mm diameter, 884 mm^3^ volume) and after cooling down the DECOTAB were removed from the moulds and stored at 7 °C. Initial DECOTAB dry mass (60 °C, 2d) was determined from a subset of ~20 DECOTAB per type.

### Consumption in laboratory microcosms

Microcosms were created by adding a layer of 1 cm pre-annealed (500 °C, 6 h) quartz sand to a glass jar (100 ml) and adding 95 ml of Dutch Standard Water (NEN 6503, 1980), consisting of 100 mg CaCl_2_∙2H_2_O, 90 mg MgSO_4_∙7H_2_O, 50 mg NaHCO_3_ and 10 mg KHCO_3_ dissolved in 1 L demineralized water (pH 8.1, hardness 210 mg L^−1^ CaCO_3_, alkalinity 1.2 meq L^−1^). We determined the consumption rates by the deposit feeder *L. variegatus* (laboratory culture) and the shredder *A. aquaticus* (collected from a nearby ditch and adjusted to the climate chamber for seven days) for each of the three DECOTAB types. We used allometric relationships for the invertebrates to standardize invertebrate biomass^44^ in order to add equal invertebrate dry mass to each microcosm. Hence six *A. aquaticus* (representative of 1350 individuals m^−2^) or twelve *L. variegatus* (2700 individuals m^−2^) were added to each microcosm at the start of the experiment. For all combinations of macroinvertebrate species and DECOTAB types we set up five replicate microcosms containing three DECOTAB. As a control for leaching and abiotic degradation, five control microcosms per DECOTAB type were included containing no detritivores. Microcosms were placed in a climate chamber (20 °C, humidity 70%, 16:8 h light:dark) and were continuously aerated. DECOTAB were collected after sixteen days, carefully washed, dried in a stove (60 °C, 2 d) and weighted.

### Consumption in ditches

Drainage ditches are a common aquatic habitat in the lowland agricultural landscape of north-western Europe and can be a significant habitat type for aquatic invertebrates^45^. Decomposition and consumption of the different DECOTAB types was studied in six ditches located in the rural area around Amsterdam (Table 1). Ditches were categorised by their macroinvertebrate community composition, being either ‘species-poor’ or ‘species-rich’ (Table 1) based on monitoring data from the local water authority Waternet and an inventory of the macroinvertebrate community composition (R. Oldenburg, MSc thesis UvA). Abiotic conditions were comparable between the two types of ditches, the only difference being a higher availability of total phosphorus in low diversity ditches (Table 1).

To facilitate retrieval in the field, we deployed cages (Ø 1 cm, height 2 cm) containing five DECOTAB of a single type closed off by either fine mesh (width 51 μm) to quantify decomposition by micro-organisms (mass loss by leaching of compounds from the agar matrix under controlled conditions was negligible), or coarse mesh (width 4 mm, three per ditch) to quantify consumption by macroinvertebrates. On 14 April 2014 at each of the six ditches (depth 0.25 to 0.65 m), three coarse mesh and one fine mesh cage per DECOTAB type were placed on the sediment alongside the bank in a randomized order at 2 m intervals. After 43 days of exposure, the cages were carefully collected from the ditches and each cage was placed in separate bag including the macroinvertebrates that had colonized the cage. DECOTAB were rinsed and subsequently dried in a stove (60 °C, 2 d) and weighted. Consumption by macroinvertebrates was calculated by subtracting mass loss of DECOTAB in fine mesh cages from mass loss of DECOTAB in the coarse mesh cages.

Since macroinvertebrate distribution in these lowland ditches is very heterogeneous^46^, we assessed the functional community composition of detritivores present on the cages. Macroinvertebrates were identified to the lowest possible taxonomic level and functional feeding type of the detritivorous macroinvertebrates (deposit feeder, scraper or shredder) was derived from the trait database^31^. Taxa with equal scores for more feeding types were assigned proportionally.

### Data analysis

Consumption rates of *A. aquaticus* and *L. variegatus* for each DECOTAB quality in the microcosms were analysed using ANOVA and post-hoc Tukey’s b test. From the field data, we analysed DECOTAB degradation (i.e. loss in coarse mesh cages), microbial decomposition (i.e. loss in fine mesh cages) and macroinvertebrate consumption (i.e. degradation minus decomposition) per DECOTAB quality and ditch species richness using two-way ANOVA and post-hoc Tukey’s b test. Data were analysed for homogeneity and transformed when necessary, data analysis was performed in IBM SPSS Statistics 20. Differences in the functional composition of invertebrate communities were tested with a Gower-based ANOSIM^47^,^48^.

## Additional Information

**How to cite this article**: Vonk, J. A. *et al*. The significance of linoleic acid in food sources for detritivorous benthic invertebrates. *Sci. Rep.* **6**, 35785; doi: 10.1038/srep35785 (2016).

## Figures and Tables

**Table 1:** Overview of the three species-poor and three species-rich ditches studied in the western part of The Netherlands.

Ditch			Macroinvertebrates Detritivores				Abiotic conditions	
#	Location	Coordinates	Species	# taxa	%	Dominant taxa	(nutrients in mg N or P L^−1^)	
1	Demmerik-1	N 52°12″08′, E 4°56″46′	poor	2	98	*Gammarus*	pH 8.01 ± 0.04	Cl 137 ± 7 mg L^−1^
4	Achtervlietpad-1	N 52°14″54′, E 5°00″10′	poor	3	100	Tubificidae	N_Kj_ 2.06 ± 0.10	NO_3_ 0.41 ± 0.04
6	Keverdijk	N 52°18″33′, E 5°05″51′	poor	2	100	*Chironomus*	P_t_ 0.14 ± 0.01	PO_4_ 0.040 ± 0.006
2	Demmerik-2	N 52°12″08′, E 4°56″48′	rich	10	96	*Gammarus/Chironomus*	pH 7.68 ± 0.07	Cl 151 ± 14 mg L^−1^
3	Demmerik-3	N 52°12″00′, E 4°56″53′	rich	10	97	*Gammarus*	N_Kj_ 2.66 ± 0.39	NO_3_ 0.34 ± 0.08
5	Achtervlietpad-2	N 52°15″01′, E 5°00″05′	rich	14	70	*Valvata/Chironomus*	P_t_ 0.33 ± 0.01	PO_4_ 0.063 ± 0.012

Coordinates, composition of the macroinvertebrate community, percentage of detritivores of the macroinvertebrate community and the dominant detritivorous taxa (initial screening) are provided and an overview of abiotic conditions (mean ± SE) derived from frequent monitoring activities by the local water authority Waternet (N_Kj_ Kjeldahl Nitrogen; P_t_ Total Phosphorus).
